# Investigation of Pneumonic Plague, Madagascar

**DOI:** 10.3201/eid2401.170760

**Published:** 2018-01

**Authors:** Michel Drancourt, Didier Raoult

**Affiliations:** MEPHI, UMR, IRD, Aix-Marseille Université, Marseille, France

**Keywords:** Yersinia pestis, bacteria, pneumonic plague, transmission, leptospirosis, ectoparasite, lice, fleas, Madagascar, Democratic Republic of the Congo, Africa

**To the Editor:** In an investigation of a pneumonic plague outbreak in Madagascar, Ramasindrazana et al. reported isolation of *Yersinia* *pestis* from 2 patients and seroconversion in 2 additional patients; these data indicated 4 (28.7%) of 14 diagnosed cases among described cases (*1*). The risk for overestimation of pneumonic plague contagion was illustrated by an outbreak in the Democratic Republic of the Congo that included cases of leptospirosis (*2*). In fact, thorough investigations in Uganda indicated that 2 index patients transmitted *Y. pestis* to only 1 caregiver each and none to 23 additional untreated close contacts (*3*). Another investigation in China showed that 3 index patients exposed 214 contacts during 3–13 days; all contacts were quarantined, and no secondary cases were reported (*4*). Transmission of *Y. pestis* by respiratory droplets requires face-to-face exposure with a coughing patient, as can occur during funerals by close contact with coughing persons who may have been exposed to the pathogen while visiting or attending the patient before he or she died. Therefore, the threat for plague epidemics fueled by pneumonic plague can be reduced by measures such as isolating patients and wearing a mask when exposure is likely (*5*).

We propose the hypothesis that only the transmission of *Y. pestis* by ectoparasites, such as lice and fleas, by close contact with infected humans can sustain outbreaks and epidemics. In plague-endemic regions, to support the appropriate management of patients and provide a rapid and accurate microbiological diagnosis, we recommend point-of-care laboratories, some of which are now operating in a few remote regions of Africa. In addition to direct diagnosis of disease in humans, direct detection of *Y. pestis* at the point-of-care in potential sources and vectors would facilitate understanding of how plague epidemics sustain.
